# Utilization of Construction Waste Recycled Powder as Filler in Asphalt Concrete

**DOI:** 10.3390/ma15165742

**Published:** 2022-08-19

**Authors:** Zemeng Guo, Zongwu Chen

**Affiliations:** 1 Zhejiang Zhongnan Construction Group Co., Ltd., Hangzhou 310052, China; 2 Faculty of Engineering, China University of Geosciences (Wuhan), Wuhan 430074, China; 3 State Key Laboratory of Subtropical Building Science, South China University of Technology, Guangzhou 510640, China

**Keywords:** construction waste, recycled powder, filler, asphalt concrete

## Abstract

Processing construction waste into aggregate and reusing it in asphalt pavement is beneficial in terms of environmental protection and resource utilization. However, recycled aggregate (RA) possesses some property defects. Therefore, RA usually needs to be strengthened by modification technologies prior to use. In order to promote the convenient and low-cost utilization of construction waste, a new method of preparing construction waste into powder and using recycled powder (RP) as asphalt filler is proposed in this research. The property defects of RA and the applicability of RP used as filler were first analyzed based on their material characteristics. Then, asphalt concrete with RP was designed according to the Superpave method, and the engineering performance of the asphalt mixture was further investigated. According to the results, we recommend the use of acidic RP in combination with other highly alkaline fillers, such as Portland cement (PC), with a suitable blending ratio of RP to PC of 1:1. Preparing asphalt concrete with filler composed of RP and PC can achieve satisfactory engineering performance.

## 1. Introduction

Roads are an important part of the transportation network, providing support for national economic development and daily travel of residents. Asphalt concrete pavement is widely used in the surface layer of roads due to its satisfactory driving comfort, low noise, easy maintenance, etc. [1]. It is mainly composed of aggregate, filler and asphalt [2,3]. Hence, the construction of asphalt pavement consumes a considerable amount of natural resources and causes many environmental problems. The development of new technologies to save natural resources [3,4,5,6,7,8,9,10,11] and protect the environment [12,13,14] is an important strategy for the sustainable development of roads. The use of solid waste, such as steel slag [4,6,7,10], reclaimed asphalt pavement (RAP) [4,15,16], construction waste [5,17,18], plastic [16,19,20], rubber [9,19,20], red mud [8], etc., to replace natural resources in the preparation of asphalt concrete has become increasingly popular, which can also help to protect the environment.

New construction and demolition of infrastructure produce a large amount of construction waste, usually comprising a mixture of steel, rebar, wood, concrete, etc. [21]. In some cases, construction waste may also contain bricks, such as in the waste from building demolition. After the sorting and crushing operations, construction waste can be processed into recycled aggregate (RA). Many studies have been conducted on cement concrete or asphalt concrete containing RA. Research results suggest that RA possesses low apparent density, high water absorption, and high crush value and abrasion value [5,11]. These property defects are believed to be related to the cement mortar and brick contained in RA, resulting poor strength and low resistance to impact load damage and abrasion. As a result, RA commonly has a negative effect on the engineering performance of asphalt concrete, especially when the RA content is high. For example, asphalt concrete with high content of RA showed low strength, poor moisture damage resistance, poor low-temperature crack resistance and unsatisfactory high-temperature deformation resistance [11,17,18].

Therefore, RA usually needs to be strengthened prior to use in asphalt concrete. Many methods can be applied to improve the properties of RA, such as organic and inorganic modifications. Zhu et al. found that sealing the surface of RA with organic silicone resin can effectively eliminate its property defects and improve the engineering performance of asphalt concrete containing RA [11]. However, the strengthening treatment process of RA is often costly and difficult to control, which makes the utilization of RA in asphalt concrete inconvenient and uneconomic. Therefore, new convenient and economical methods for the utilization of construction waste are needed. The use of construction waste as a filler for asphalt concrete is one such method [2,22,23,24]. With the exception of aggregate, filler is also indispensable, although its content in asphalt concrete is usually less than 10% [8,25]. As China faces the considerable task of road construction, if asphalt concrete containing construction-waste-based filler can obtain satisfactory engineering performance, it could represent a simple method for the reuse of construction waste.

Many attempts have been made to use construction waste as asphalt concrete filler, such as in the form of glass powder (GP) [2], brick powder (BP) [2,22,24], recycled fine aggregate powder (RFAP) [23], etc. The effects of such fillers on the performance of asphalt concrete share similarities but also differ in some aspect. GP, BP and RFAP all showed positive effects on the improvement of high-temperature deformation resistance of asphalt mastic or asphalt concrete [2,22,23,24], with different results with respect to other performance measures. For example, Choudhary et al. suggested that GP and BP perform poorly with respect to maintaining the moisture resistance of asphalt concrete [2]. Chen et al. found that RFAP can result in asphalt concrete with satisfactory moisture stability [23] as a result of its mineralogical and chemical composition. Asphalt concretes with fillers containing calcium-based minerals, such as RFAP, have been proven to achieve improved adhesion performance and reduced moisture susceptibility relative to asphalt concretes with silica-based fillers, such as GP and BP [2]. Therefore, limestone powder (LP) is the most frequently used filler in asphalt concrete. LP is easy to obtain, and it is also an alkaline calcium-based filler (CaCO_3_). Alkaline calcium-based fillers are believed to improve the compatibility of weak acid bitumen and filler particles in asphalt mastic and increase the bonding performance between asphalt mastic and aggregate [6].

In this research, we used construction waste resulting from the demolition of concrete pavement. Compared with other types of construction waste, its components are simpler. It consists entirely of crushed stone aggregate and cement mortar. Waste concrete blocks were first crushed and ground into powder; then, the recycled powder (RP) was used as asphalt filler. The acidity or alkalinity of RP is determined by the crushed stone aggregate, although cement mortar is highly alkaline. This is because the amount of crushed stone aggregate in waste concrete is much higher than that of cement mortar. Crushed stone aggregate can be divided into three categories according to composition: acidic, neutral and alkaline aggregates. If RP produced from waste concrete containing acidic crushed aggregate can be used to produce asphalt concrete with satisfactory performance by taking some appropriate technical measures, RP produced from waste concrete containing neutral and alkaline crushed aggregates can also achieve the goal. Accordingly, this study used the RP produced by processing waste concrete containing acidic crushed stone aggregate as the research object. According to previous research, when acidic materials are incorporated into asphalt concrete, highly alkaline fillers, such as hydrated lime [26] and Portland cement [27], can strengthen the bonding performance of the concrete. Therefore, this strategy was adopted in the present research.

Based on the above information, in the present study, we (1) analyzed the unsuitability of directly using RA in asphalt concrete and the applicability of RP as an asphalt filler according to its material characteristics. (2) Then, asphalt concretes were designed according to the Superpave method, and the appropriate blending ratio of RP and highly alkaline filler was determined based on the test results of the engineering performance of asphalt concretes, including moisture damage resistance, high-temperature deformation resistance and low-temperature crack resistance.

## 2. Raw Materials and Experimental Methods

### 2.1. Raw Materials

The raw materials used in this research included two types of coarse aggregates, namely limestone and recycled aggregate (RA); a limestone fine aggregate; three types of fillers, namely limestone powder (LP), recycled powder (RP) and Portland cement (PC); and an SBS-modified asphalt binder. Aggregates, LP and RP were provided by a local crushed stone factory in Hangzhou, China. RA and RP were obtained by crushing and grinding waste concrete blocks, respectively. The waste concrete blocks mainly consisted of granite crushed stone and cement mortar. PC was provided by the Zhejiang Southern Cement Co., Ltd., Hangzhou, China. The SBS-modified asphalt was from the Zhejiang Transportation Resources Investment Co., Ltd. Asphalt Technology Branch, Hangzhou, China.

According to the Chinese technical specification [28], the basic technical properties of raw materials should meet certain requirements before they can be used. The basic technical properties of aggregate, fillers and SBS-modified asphalt were tested according to the Chinese standard methods [29,30], and results are listed in Table 1, Table 2 and Table 3. The basic technical properties of RA and limestone coarse aggregate will be compared and analyzed in Section 3.1, so these results are not shown here. Table 1, Table 2 and Table 3 show that all the tested basic technical properties of the used limestone fine aggregate, fillers (LP, RP and PC) and SBS-modified asphalt met the requirements of the Chinese specification [28]. Table 2 shows that the particle gradations of LP, RP and PC do not differ significantly. RP is slightly finer than LP and PC, and LP is the coarsest of the three investigated fillers.

### 2.2. Experimental Methods

#### 2.2.1. Unsuitability Analysis of Directly Using RA in Asphalt Concrete

The basic technical properties of RA and common limestone coarse aggregate, including apparent specific gravity, water absorption, crush value and abrasion value, were first compared to reveal the property defects of RA. Then, a JSM-IT300 scanning electron microscope (SEM) from JEOL, Japan was used to observe the micromorphology of RA, the reasons for the property defects were analyzed based on the characteristics of the SEM images.

#### 2.2.2. Applicability Analysis of RP as an Asphalt Filler

The mineral phases and chemical compositions of RP and LP were analyzed by a D8 Advance X-ray diffraction (XRD) from Bruker, Germany, and a Zetium X-ray fluorescence (XRF) from PANalytical, Netherlands, respectively. The XRD data were analyzed by means of Jade software with embedded PDF data from the International Centre for Diffraction Data (ICDD). A scheme suitable for the utilization of RP in asphalt concrete was proposed after comparing the mineral phases and chemical compositions of RP to those of LP.

#### 2.2.3. Design of Asphalt Concretes

**Figure 1:**
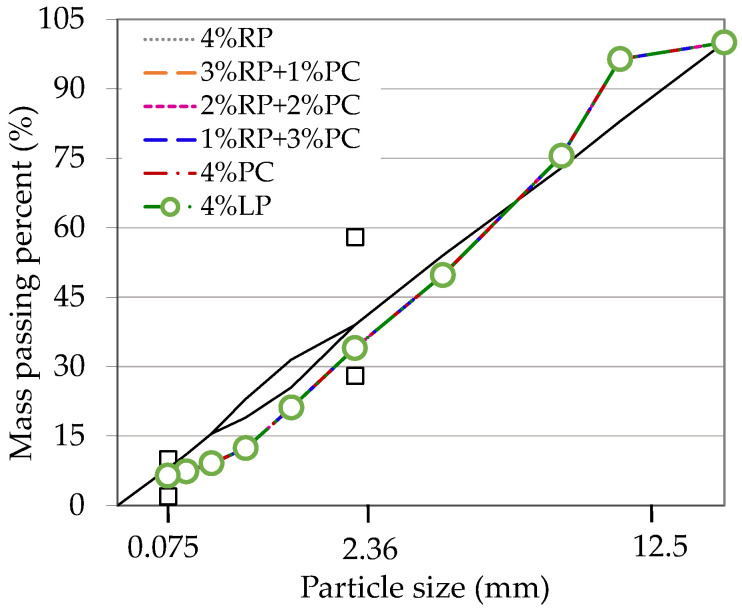
Hybrid gradations used in this research.

According to the analysis results presented in Section 2.2.2, we suggested the use of RP, together with highly alkaline powders. Alkaline PC was adopted in this research. As shown in Table 4, a total of five asphalt concretes were designed in order to determine the effect of the blending ratio of RP and PC on the engineering performance of asphalt concrete. The Superpave design method was used, with contents of coarse aggregate, fine aggregate and filler of 55%, 41% and 4%, respectively. The hybrid gradations of all asphalt concretes are shown in Figure 1, with similar values across all samples.

#### 2.2.4. Engineering Performance Evaluation of Asphalt Concretes

*Moisture damage resistance*. The moisture damage resistance of asphalt concretes was investigated by a retained Marshall stability (RMS) test and tensile strength ratio (TSR) test according to Chinese technical specifications T0709 and T0729, respectively [29]. Tested samples with a diameter of 100 mm and a thickness of 63.5 mm were prepared by coring and cutting large cylindrical specimens compacted in a Superpave gyratory compactor. The prepared samples were equally divided into a blank group and several conditioned groups.

In the RMS test, each conditioned group was first immersed in a water bath at 60 °C for varying durations; then, the Marshall stability of each sample in the blank group and the conditioned groups was measured. The RMS can be further computed by Equation (1). A higher RMS value is associated with reduced loss of Marshall stability and improved resistance of asphalt concrete to hot water damage.

(1) $$\mathrm{RMS}=\frac{S_{i}}{S_{0}}\times 100\%$$

where *S*_0_ is the average Marshall stability of all samples in the blank group, and *S_i_* is the average Marshall stability of all samples in each conditioned group after immersion in a hot water bath for *i* h.

In the TSR test, each conditioned group was subjected to freeze–thaw damage for a varying number of cycles; then, the splitting strength of each sample in the blank group and conditioned groups was measured. One freeze–thaw cycle consists of freezing at −18 °C for 16 h, followed by thawing in a 60 °C water bath for 24 h. The TSR can be further computed by Equation (2). A higher TSR value indicates reduced loss of the splitting strength and improved resistance of asphalt concrete to freeze–thaw cycle damage.

(2) $$\mathrm{TSR}=\frac{SS_{h}}{SS_{0}}\times 100\%$$

where *SS*_0_ is the average splitting strength of all samples in the blank group, and *SS_h_* is the average splitting strength of all samples in each conditioned group after freeze–thaw damage for *h* cycles.

*High-temperature deformation resistance*. The high-temperature deformation resistance of asphalt concrete was evaluated by the wheel tracking test according to Chinese technical specification T0719 [29]. Slab samples with a 300 mm length, 300 mm width and 50 mm height compacted by a wheel roller were used. Assembling slab sample to a wheel tracking device with a steel wheel with a width of 50 mm. The wheel rolled back and forth along the center line of the sample surface at 60 °C and at set pressure for 1 h. The dynamic stability can be computed by Equation (3).

(3) $$\mathrm{DS}=\frac{15\times s}{l_{2}-l_{1}}$$

where DS is the dynamic stability (pass/mm); *s* is the wheel speed (42 pass/min); and *l*_1_ and *l*_2_ are the rutting deformation depths of the slab sample when the test time reaches 45 min and 60 min, respectively (mm).

*Low-temperature crack resistance*. The low-temperature crack resistance of asphalt concrete was evaluated by three-point bending beam test according to Chinese technical specification T0728 [29]. The tested beam samples with 250 mm length, 30 mm width and 35 mm height were prepared by cutting the asphalt concrete slabs according to the same method used in the high-temperature deformation resistance evaluation. The bending beam test was carried out with a mechanical testing machine. The indenter was loaded in the middle of the sample to force it to deform until the failure occurred. The test temperature was −10 °C, and the deformation speed of the beam sample was 50 mm/min. The flexural strength, strain and stiffness modulus can be computed by Equations (4)–(6).

(4) $$\sigma (t)=\frac{3LP(t)}{2bh^{2}}$$

(5) $$\varepsilon (t)=\frac{6hd(t)}{L^{2}}$$

(6) $$S_{f}=\frac{\sigma_{\max}}{\varepsilon_{f}}$$

where *σ*(*t*) is the real-time flexural strength of the sample (MPa); *P*(*t*) is the real-time load borne by the sample during the bending process (kN); *l*, *b* and *h* are the spanning length, width and height of the sample, respectively (mm); *ε*(*t*) is the real-time strain of the sample (με); *d*(*t*) is the real-time vertical deflection of the sample (mm); *S_f_* is the flexural stiffness modulus of the sample at failure (MPa); *σ*_max_ is the flexural tensile strength of the sample at failure (MPa); and *ε_f_* is the flexural tensile strain of the sample at failure (με).

## 3. Results and Discussion

### 3.1. Unsuitability Analysis of Directly Using RA in Asphalt Concrete

**Figure 2:**
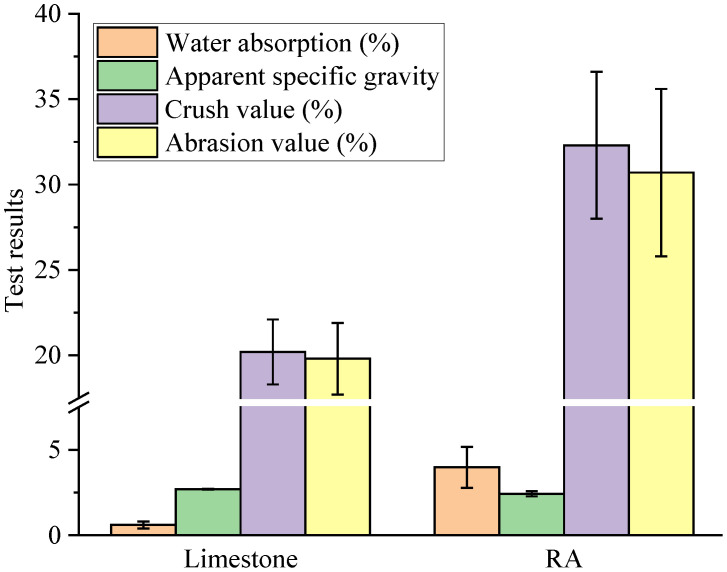
Basic technical properties of RA and limestone aggregate.

Four basic technical properties with significant differences between RA and limestone are shown in Figure 2. Compared with limestone aggregate, RA presented with lower apparent specific gravity, higher water absorption, higher crush value and higher abrasion value. Specifically, the apparent specific gravity of RA was 2.426, which is 10.1% less than that of limestone aggregate. The water absorption, crush value and abrasion value of RA were 3.98%, 32.3% and 30.7%, respectively, which is 563.3%, 59.9% and 55.1% higher those of limestone aggregate, respectively. According to the Chinese technical specification for construction highway asphalt pavement [28], the apparent specific gravity, water absorption, crush value and abrasion value of aggregates for high-grade highways should be more than 2.5, and less than 3%, 28% and 30%, respectively. Therefore, these four basic technical properties of RA do not meet the requirements of the Chinese technical specification, and RA cannot be directly used in the construction of asphalt pavement as aggregate.

**Figure 3:**
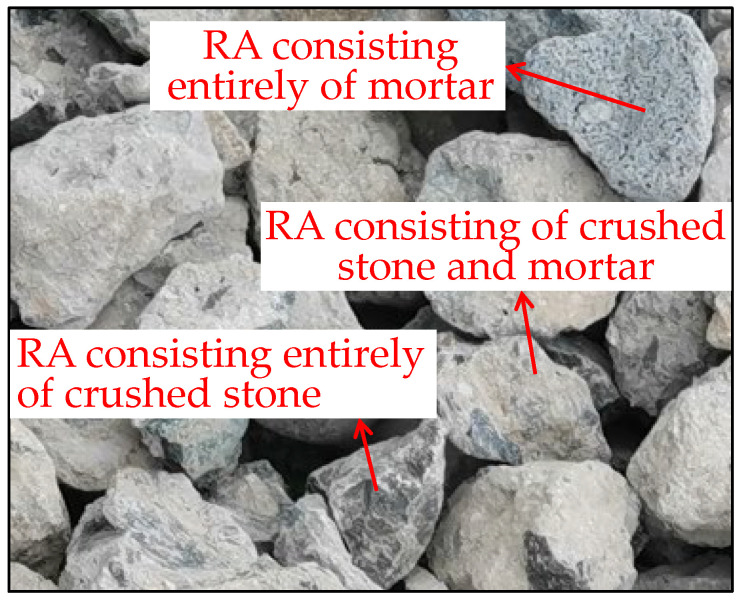
Macroscopic appearance of RA.

The property deficiencies are related to the macro- and microstructure of RA. Crushed stone aggregate and cement mortar are the main components of concrete. Therefore, the components of RA particles produced by crushing waste concrete blocks are variable and can be distinguished according to their appearance. The macroscopic appearance of RA is presented in Figure 3, showing three kinds of RA particles with varying morphologies: particles consisting of crushed stone and mortar, particles consisting entirely of mortar and particles consisting entirely of crushed stone. Furthermore, the mortar contains many macroscale pores.

**Figure 4:**
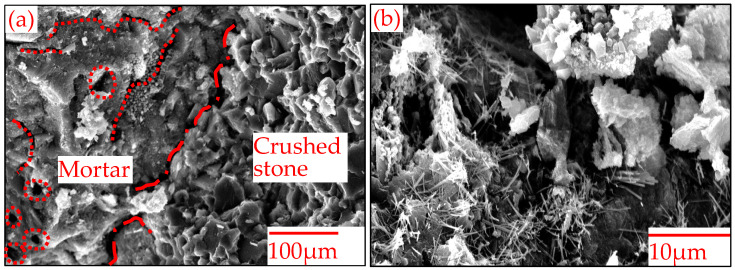
Micromorphologies of RA: (**a**) Contact interface between crushed stone and mortar and (**b**) mortar.

The micromorphology of RA was further analyzed by SEM. Particles consisting of crushed stone and mortar were used because the morphologies of crushed stone, mortar and their contact area can be observed simultaneously. The SEM images are displayed in Figure 4. Figure 4a shows that the micromorphology of mortar differs obviously from that of crushed stone. The former showed many cracks and pores, and the later showed obvious graininess, with a coarser and denser texture. In mortar layer, cement hydration and carbonization products, such as acicular ettringite, flocculent C-S-H gel and prismatic calcite, were further found in some regions under a higher SEM magnification, as shown in Figure 4b. In addition, compared with microcracks in cement mortar, larger microcracks on the contact interface between crushed stone and mortar were observed. Unlike the macropores and micropores within mortar that are formed during cement hydration, microcracks are mainly formed during the preparation of recycled aggregate by crushing waste concrete blocks.

The macropores and micropores within mortar and the microcracks on the interface between crushed stone and cement result in RA with high water absorption ability (563.3% higher water absorption than limestone aggregate), low density (10.1% lower apparent specific gravity than limestone aggregate) and poor mechanical properties (59.9% higher crush value and 55.1% higher abrasion value than limestone aggregate) relative to limestone aggregate.

The poor properties of RA make it unsuitable for direct use in asphalt concrete as aggregate. Technical measures are usually required to strengthen the properties of RA, such as an organic modification method [11]. However, strengthening treatment increases the complexity of the RA preparation process, resulting in increased cost. Therefore, there is an urgent need to develop more convenient and low-cost utilization methods for waste concrete. The feasibility of using RP in asphalt concrete as filler is discussed in the following section.

### 3.2. Applicability Analysis of RP as an Asphalt Filler

**Figure 5:**
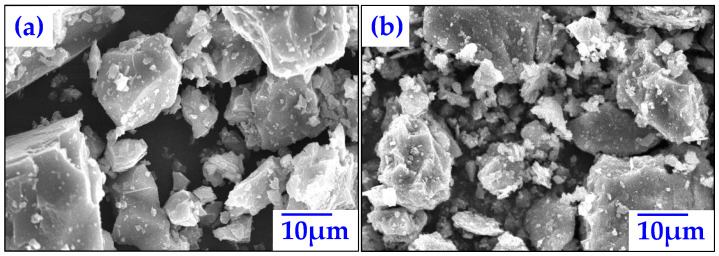
SEM images: (**a**) LP and (**b**) RP.

The micromorphologies of LP and RP are shown in Figure 5. LP and RP are both composed of numerous particles with varying microscopic sizes. The large particles in LP and RP presented very similar angular features and rich angularity. Compared with LP, large particles in RP possessed a coarser surface texture, with more small floccules. The floccules are believed to be related to the hydration products of cement. The filler mainly forms asphalt mortar with asphalt to play a cementing role in asphalt concrete; if RP is used as filler, coarser surface texture and rich floccules are beneficial for the interaction between RP and asphalt. Theoretically, the stability of RP asphalt mastic is improved relative to that of LP asphalt mastic.

**Figure 6:**
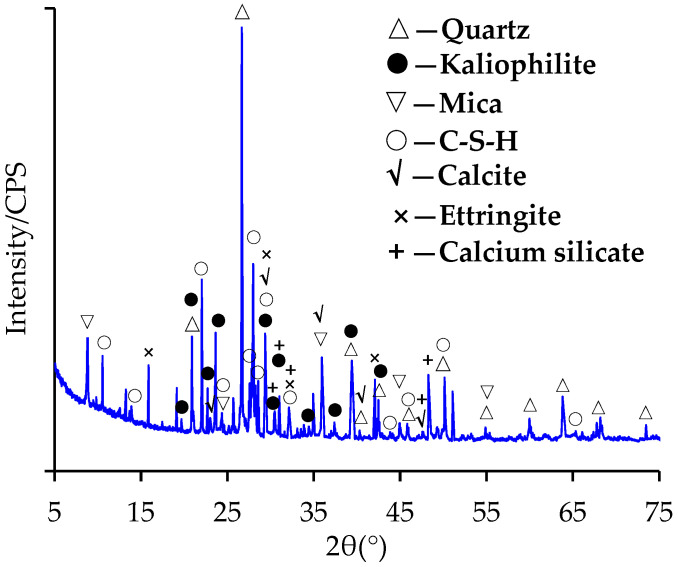
XRD results of RP.

**Figure 7:**
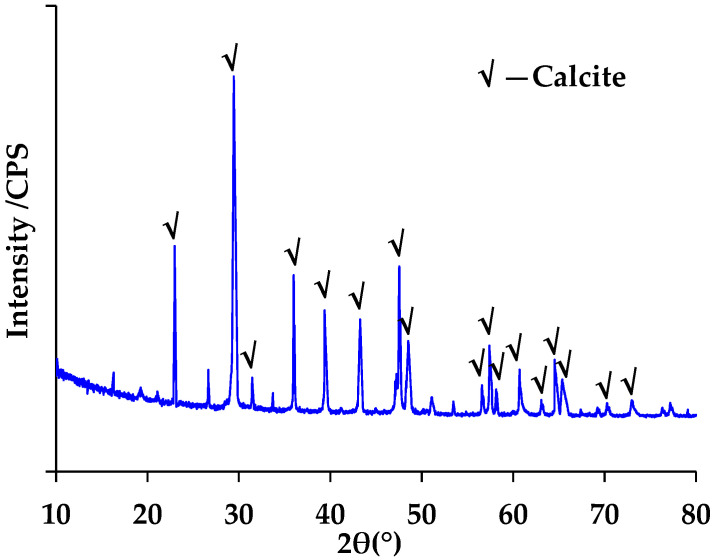
XRD results of LP.

The XRD analysis results of RP and LP are shown in Figure 6 and Figure 7, respectively. Figure 6 indicates that the minerals in RP were mainly quartz, kaliophilite, mica, C-S-H, calcite, ettringite and calcium silicate. These results are similar to those reported in previous research. Chen et al. also detected calcite and quartz in RP from fine recycled aggregate [23]. The RP used in this research presented with more mineral types, mainly due to the difference in the components of construction waste. The waste concrete used in this research was composed of granite crushed stone and cement mortar. The granite crushed stone was mainly composed of quartz, kaliophilite and mica. The C-S-H, ettringite, calcite and calcium silicate were contributed by the hydration and carbonization of cement. It is well known that the LP contains calcite, as shown in Figure 7.

XRD results can explain the chemical composition results obtained with XRF. The main chemical compositions of RP and LP are listed in Table 5. The chemical composition of quartz was SiO_2_. The kaliophilite and mica mainly contained K_2_O, Al_2_O_3_ and SiO_2_. The hydration and carbonization products of cement, including C-S-H gel, calcite, ettringite and calcium silicate, contained large amounts of CaO, SiO_2_, SO_3_, etc. According to Table 5, except for these mentioned oxides, the content of Fe_2_O_3_ in RP was also relatively high, which may be related to the tetracalcium ferric aluminate contained in cement. Therefore, SiO_2_, CaO, Al_2_O_3_, Fe_2_O_3_, K_2_O and SO_3_ were the main chemical compositions of RP in oxide form. The main chemical composition of LP was CaO according to XRF analysis results. In addition, RP and LP displayed large loss on ignition (LoI), mainly caused by the decomposition of CaCO_3_ during XRF analysis.

The high content of SiO_2_ in RP makes it strongly acidic, which is not conducive to in asphalt concrete as a filler. Therefore, RP cannot be used alone as a filler in asphalt concrete. As previously stated, using highly alkaline powder to partially or completely replace filler is a common technical measure employed to strengthen the bonding performance between various components in asphalt concrete when acidic materials are involved [3,26,27]. Therefore, there is a need to use RP in combination with a highly alkaline filler. PC (Portland cement) is frequently adopted in China to improve the performance of asphalt concrete. In order to adapt to engineering application scenarios, in this research, we also used highly alkaline PC in combination with RP. The appropriate blending ratio of RP and PC was determined based on the following results of the engineering performance of asphalt concrete.

Given that RP produced from waste concrete containing acidic crushed aggregate can result in satisfactory performance of asphalt concrete when used in combination with highly alkaline PC, RPs produced from waste concrete containing neutral or alkaline crushed aggregate can achieve similar results. This is the original intention behind the use of waste concrete blocks containing granite crushed stone to prepare RP in this research.

### 3.3. Engineering Performance Evaluation of Asphalt Concrete

#### 3.3.1. Moisture Damage Resistance

**Figure 8:**
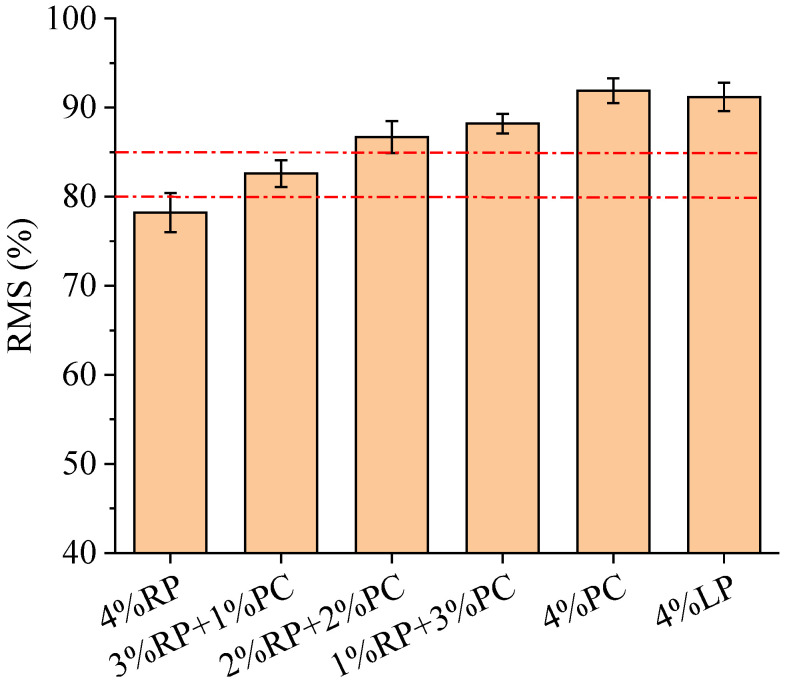
RMS results of asphalt concretes.

The RMS results of asphalt concretes are displayed in Figure 8. The RMS of asphalt concrete with 4%RP was the lowest among the six tested asphalt concretes. With the continuous increase in the proportion of PC to replace RP, the RMS of asphalt concrete gradually increased. After the RP was fully replaced by PC, the RMS of asphalt concrete increased from 78.2% to 91.9%, indicating that PC improved the hot water damage resistance of asphalt concrete. However, the use of high PC content is not recommended for two reasons. One is that the difference of RMS between asphalt concrete with 4%LP and asphalt concrete with 4%PC was quite small, and the other is that the use of high PC content increases the cost. According to the requirements of the Chinese technical specification [28], the RMS of asphalt concrete prepared with modified asphalt binder in wet and humid regions should be not less than 85% or 80% in semi-dry and dry regions. With the exception of the two asphalt concretes with fillers composed of 4% RP and 3% RP+1%PC, respectively, the RMS values of the other four asphalt concretes all meet these requirements. Considering the need to use as much RP and as little PC as possible, filler composed of 2%RP+2%PC is recommended for use in asphalt concrete.

**Figure 9:**
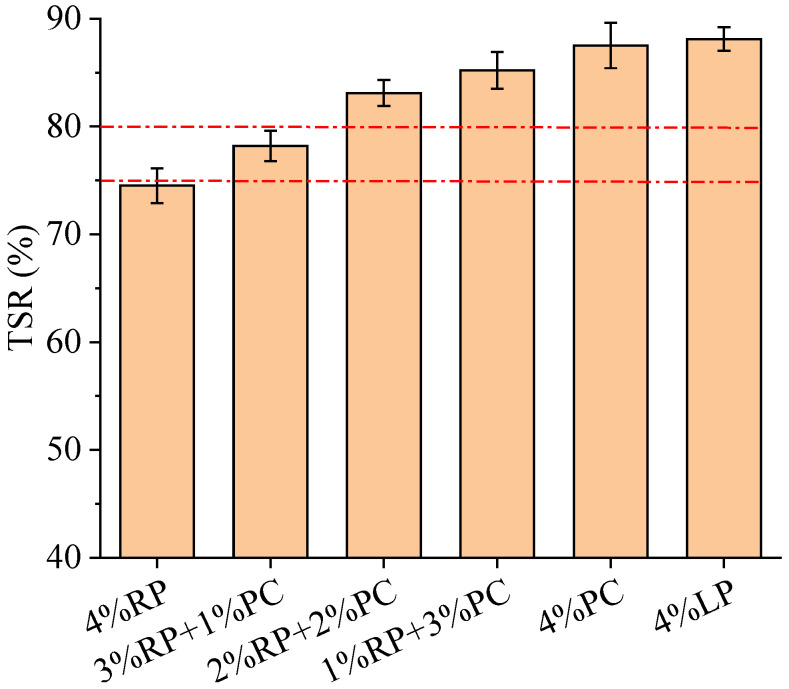
TSR results of asphalt concretes.

The TSR results of asphalt concretes shown in Figure 9 indicate similar variation rules to those of RMS, with a continuous increase in the proportion of PC to replace RP. The TSR of asphalt concrete with 4%RP was the lowest among the six tested asphalt concretes. With a continuous increase in PC in the composite filler system, the TSR of asphalt concrete gradually increased. After the RP was fully replaced by PC, the TSR of asphalt concrete increased from 74.5% to 87.5%, representing an improvement of 17.5%, meaning that the contribution of PC to the freeze–thaw damage resistance of asphalt concrete was also outstanding. According to the requirements of the Chinese technical specification [28], the TSR of asphalt concrete prepared with modified asphalt binder in wet and humid regions should be not less than 80% or 75% in semi-dry and dry regions. Figure 9 shows that asphalt concretes with fillers composed of 2%RP+2%PC, 1%RP+3%PC, 4%PC and 4%LP, respectively, can satisfy these requirements. Figure 9 also shows that 4%PC achieved slightly worse performance in terms of maintaining the TSR of asphalt concrete relative to 4%LP. Therefore, in order to save PC and use as much RP as possible, filler composed of 2%RP+2%PC is recommended for use in asphalt concrete to improve the freeze–thaw damage resistance.

RMS and TSR results suggest that the use of RP alone cannot provide asphalt concrete with satisfactory moisture damage resistance. The combined use of RP and highly alkaline PC can improve moisture stability. This conclusion is inconsistent with some previous research findings. Chen et al. stated that asphalt concrete with RP from recycled fine aggregate showed good moisture damage resistance [23]. Hasan and Ramin found that the introduction of RP from waste bricks can improve the moisture damage resistance of asphalt concrete to varying degrees [22]. The inconsistent conclusions may be related to the mineralogical and chemical composition of different RPs. Calcium-based filler can improve the adhesion performance and moisture damage resistance of asphalt concrete relative to silica-based filler [2]. In this research, an acidic silica-based RP filler was used.

#### 3.3.2. High-Temperature Deformation Resistance

**Figure 10:**
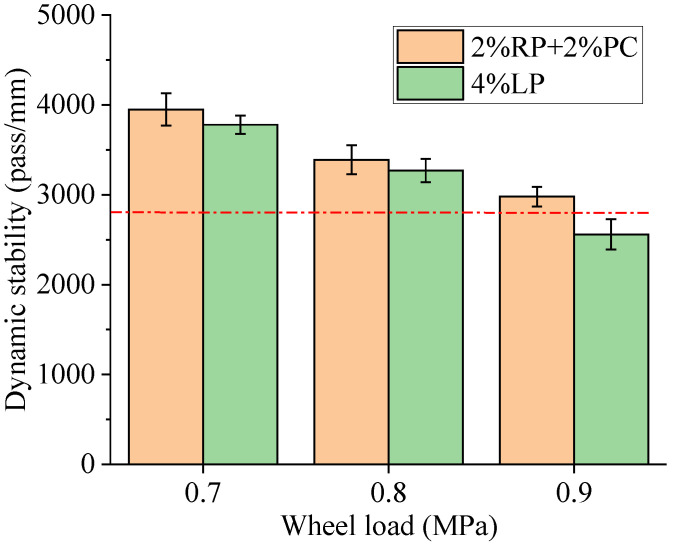
Dynamic stability results of asphalt concretes.

**Figure 11:**
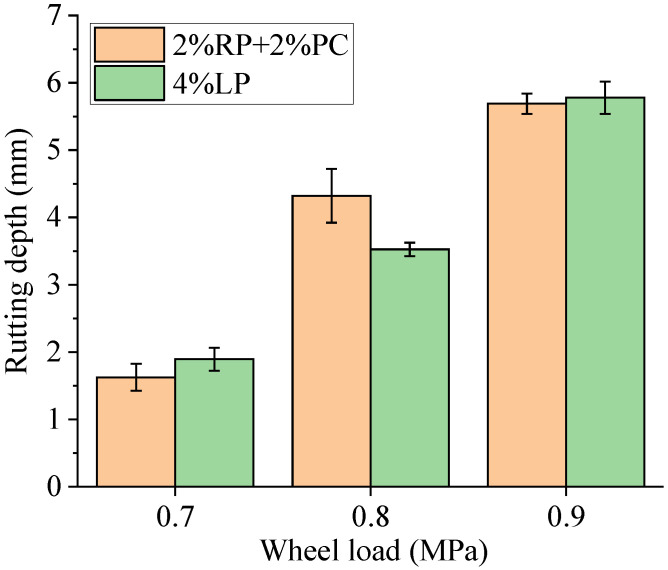
Rutting depths of asphalt concretes.

The rutting test results of asphalt concretes are shown in Figure 10 and Figure 11. Figure 10 shows that for asphalt concrete with filler composed of 2%RP+2%PC or asphalt concrete with 4%LP filler, their dynamic stabilities were quickly decreased with increased wheel load. The dynamic stability of the former formulation dropped from 3952 pass/mm to 2981 pass/mm when the wheel load increased from 0.7 MPa to 0.9 MPa, representing a reduction of 24.6%. The dynamic stability of the latter formulation dropped from 3778 pass/mm to 2560 pass/mm, representing a reduction of 32.2%. These results indicate that the high-temperature deformation resistance of asphalt concrete is sensitive to traffic load, and the high-temperature stability of asphalt concrete with filler composed of 2%RP+2%PC was better than that with 4%LP filler, especially under a higher wheel load (0.9 MPa).

According to the most rigorous requirement of the Chinese technical specification [28], the dynamic stability of asphalt concrete prepared with modified asphalt binder for hot summer regions should be not less than 2800 pass/mm under a wheel load of 0.7 MPa. Figure 10 shows that the dynamic stability of asphalt concrete with filler composed of 2%RP+2%PC was still greater than 2800 pass/mm, even under a wheel load of 0.9 MPa. Therefore, dynamic stability results indicate that the filler composed of 2%RP+2%PC performed better in terms of the high-temperature stability of asphalt concrete relative to the common LP filler.

The variation rules of rutting depths of asphalt concretes shown in Figure 11 differed slightly from those of the dynamic stability. The rutting depths of asphalt concrete with filler composed of 2%RP+2%PC were slightly smaller than those of asphalt concrete with 4%LP filler under wheel loads of 0.7 MPa and 0.9 MPa. While the rutting depth of the former was 22.6% deeper than that of the later under a wheel load of 0.8 MPa, possibly because the compaction degrees of slab samples used to test the high-temperature deformation resistance of asphalt concrete with filler composed of 2%RP+2%PC under a wheel load of 0.8 MPa were not exactly the same. This phenomenon occurred easily when manually filling the mixture to prepare samples, resulting in variable deformation depths between samples, as supported by the larger error bar corresponding to the rutting depth of asphalt concrete with filler composed of 2%RP+2%PC under a wheel load of 0.8 MPa shown in Figure 11. These samples with slightly lower compaction degrees were further compacted in the early stage of the wheel tracking test, and the process can be finished in a very short time, at which point the samples begin the stable deformation stage. According to Figure 10, the error bar size corresponding to the dynamic stability of asphalt concrete with filler composed of 2%RP+2%PC under a wheel load of 0.8 MPa was not significantly larger than others, suggesting that the dynamic stability was less affected and the deformation speed of further compacted samples was similar to that of others during the stable deformation stage. Therefore, wheel tracking test results indicate that using dynamic stability to determine the high-temperature performance of asphalt concrete was more reliable than the final rutting depth.

#### 3.3.3. Low-Temperature Crack Resistance

**Figure 12:**
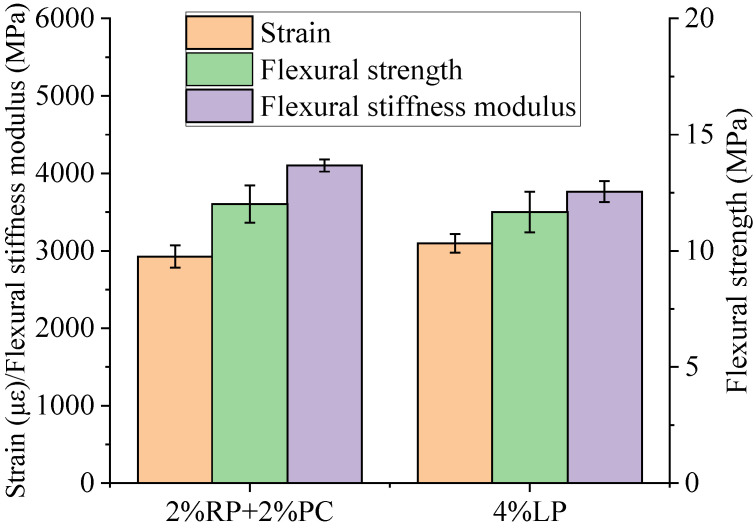
Three-point bending beam test results of asphalt concretes.

The low-temperature three-point bending beam test results are listed in Figure 12, showing that the low-temperature flexural strains of these two asphalt concretes with fillers composed of 2%RP+2%PC and 4%LP were 2926 με and 3095 με, respectively. According to the requirements of the Chinese technical specification [28], the low-temperature flexural strain of asphalt concrete prepared with modified asphalt binder for winter severe cold regions and winter cold regions should be not less than 3000 με and 2800 με, respectively. Hence, although the strain of asphalt concrete with filler composed of 2%RP+2%PC was slightly lower, it can still meet the utilization requirement for winter cold regions.

Unlike the strain, the flexural strength and flexural stiffness modulus of asphalt concrete with filler composed of 2%RP+2%PC were both higher than those of asphalt concrete with filler composed of 4%LP. In particular, the flexural strength and flexural stiffness modulus of the former were 29.2% and 8.9% higher, respectively, than those of the latter. Therefore, compared with asphalt concrete with common LP filler, the strength and stiffness modulus suggest that asphalt concrete with filler composed of 2%RP+2%PC has better low-temperature load-bearing capacity, although its strain was slightly lower.

## 4. Conclusions

In this work, waste concrete mainly composed of granite crushed stone aggregates and cement mortar were processed into powder, and the feasibility of using this recycled powder (RP) as a filler in asphalt concrete was evaluated. According to the discussed results, the following conclusions can be drawn:

1. Compared with common limestone aggregate, recycled aggregate (RA) presented 10.1% lower apparent specific gravity, 563.3% higher water absorption, 59.9% higher crush value and 55.1% higher abrasion value. These four basic technical properties do not meet the requirement of the Chinese technical specification. Therefore, RA cannot be directly used in asphalt pavement as aggregates, and convenient and low-cost utilization methods are needed for waste concrete.
2. Compared with common limestone powder (LP), RP showed a coarser microtexture and contained rich floccules. These characteristics are beneficial for the interaction between RP and asphalt in asphalt mastic. The high SiO_2_ content mainly contributed by quartz minerals makes RA strongly acidic. Acidic mineral materials are not suitable for asphalt concrete. Therefore, RP is recommended for use in combination with other highly alkaline fillers.
3. RMS and TSR results suggest that the appropriate blending ratio of RP and highly alkaline Portland cement (PC) in a hybrid filler system is 1:1. Preparing asphalt concrete with hybrid filler composed of 2%RP+2%PC can result in satisfactory moisture damage resistance, high-temperature deformation resistance and low-temperature crack resistance.

## Figures and Tables

**Table 1:** Basic technical properties of limestone fine aggregate.

Property	Results	Requirement
Apparent specific gravity	2.711	≥2.5
Water absorption (%)	1.3	≤2
Fine aggregate angularity (%)	58	≥30
Sand equivalent (%)	66	≥60

**Table 2:** Basic technical properties of the three investigated fillers.

Property		LP	RP	PC	Requirement
Specific gravity (g/cm^3^)		2.715	2.588	3.011	≥2.5
Percent passing (%)	0.6 mm	100	100	100	100
	0.15 mm	92.1	95.2	93.7	90–100
	0.075 mm	86.2	88.5	88.1	75–100

**Table 3:** Basic technical properties of SBS-modified asphalt binder.

Property	Results	Requirement
Softening point (°C)	80.2	≥60
Penetration (25 °C; 0.1 mm)	58.8	40–60
Ductility (5 °C; cm)	37.8	≥20
Viscosity (135 °C; Pa·s)	0.955	≤3
Elasticity résumé (25 °C; %)	77	≥75

**Table 4:** Mass blending ratio of mineral raw materials in asphalt concrete.

Asphalt Concrete	Coarse Aggregate	Fine Aggregate	Filler
1	55% limestone	41% limestone	4%RP
2			3%RP+1%PC
3			2%RP+2%PC
4			1%RP+3%PC
5			4%PC
6			4%LP

**Table 5:** Main chemical compositions of RP and LP.

Chemical Composition	RP (%)	LP (%)
SiO_2_	52.36	1.03
Al_2_O_3_	10.56	0.85
CaO	12.72	50.65
Fe_2_O_3_	5.72	0.45
MgO	1.12	0.46
K_2_O	4.95	0.21
SO_3_	3.48	0.17
LoI	7.85	42.21

## Data Availability

Not applicable.
